# Establishing diagnostic training programs in resource-poor settings: The case of Sierra Leone

**DOI:** 10.4102/ajlm.v9i1.889

**Published:** 2020-06-15

**Authors:** Lance D. Presser

**Affiliations:** 1 Global Engagement Program, MRIGlobal, Gaithersburg, Maryland, United States

## Introduction

### Outbreak and initial response

The West Africa Ebola virus disease (EVD) epidemic in 2014–2016 resulted in at least 28 652 total cases (15 261 laboratory confirmed), of which at least 11 325 were fatal (case fatality rate ~40%).^1^ During the epidemic, most of the cases were concentrated in Liberia, Guinea, and Sierra Leone, with some cases exported to the United States, Nigeria, Mali, and other countries around the world.^2^

**FIGURE 1:**
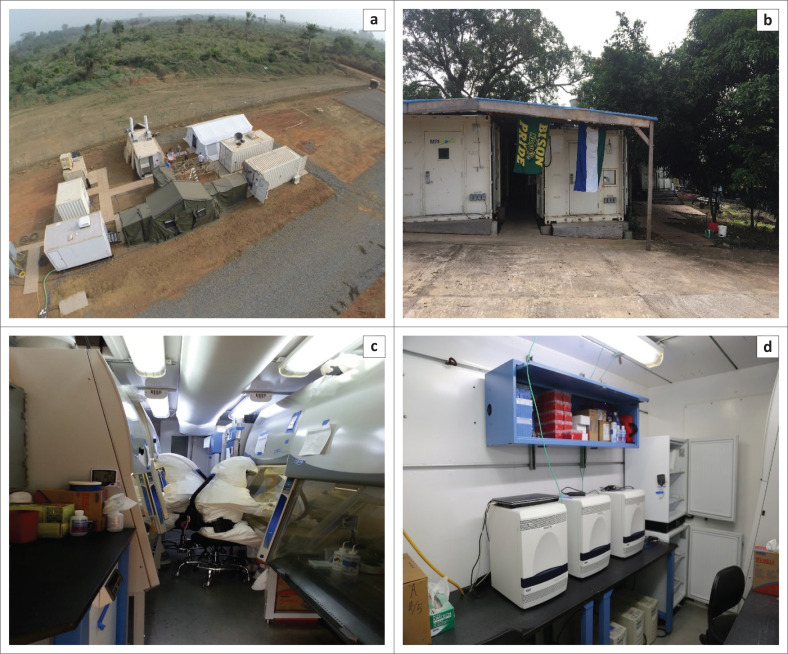
MRIGlobal mobile diagnostic laboratory in Sierra Leone. (a) Setup of the mobile diagnostic laboratory in Moyamba, Sierra Leone, January 2015 (aerial view). (b) Setup of the mobile diagnostic laboratory at the Sierra Leone Central Public Health Reference Laboratory in Lakka, Freetown (moved in April 2015, photograph is from November 2016). (c) Interior of extraction laboratory unit. (d) Interior of molecular diagnostics laboratory unit.

Cases of EVD began appearing in Sierra Leone in May 2014. MRIGlobal first deployed to Sierra Leone in January 2015 and has maintained a presence in the country ever since, resulting in numerous deployments for diagnostics, engineering and, now, training teams (Figure 1). MRIGlobal provided assistance to the government of Sierra Leone and international partners to implement diagnostic testing, training courses, and other outbreak-related activities (Table 1). MRIGlobal supported the national and district EVD surveillance databases and provided data for EVD surveillance, contact tracing, case investigation, et cetera. It trained staff and offered support to members of the National Rapid Response Team at the Sierra Leone Central Public Health Reference Laboratory (CPHRL). It established, managed, and staffed EVD testing laboratories in both Sierra Leone and Guinea (Figure 1). Initially, the mobile laboratory was in Moyamba, Sierra Leone, but in April 2015 it moved to Lakka in Freetown, Sierra Leone, on the same grounds as the CPHRL.

**TABLE 1:** Number of confirmed, probable, and suspected Ebola virus disease cases, number of deaths and number of MRIGlobal staff deployments during the Ebola virus disease epidemic – Guinea, Liberia, and Sierra Leone, March 2014 – September 2016.

Characteristic	Guinea	Liberia	Sierra Leone
Date of first confirmed case	March 2014	March 2014	May 2014
No. of confirmed, probable and suspected cases	3811	10 675	14 124
No. of deaths	2543	4809	3956
No. of MRIGlobal staff deployments	49	2	67

### International partner training programmes in Sierra Leone

Numerous international partners developed programmes in Sierra Leone during and after the West Africa EVD outbreak. The United States Centers for Disease Control and Prevention (CDC), China CDC (CCDC), Association of Public Health Laboratories (APHL), World Health Organization, Public Health England (PHE) and a number of other organisations developed and conducted a variety of training events. The following is a brief summary of the training activities hosted by international partners in Sierra Leone.

The APHL works to build laboratory systems in the United States and globally. Its international work focuses on building national laboratory systems and expanding access to quality diagnostic testing systems. During the outbreak, APHL, in partnership with MRIGlobal, trained 26 National Rapid Response Team laboratory scientists and provided consultation regarding the National Strategic Plan of Sierra Leone’s Ministry of Health and Sanitation (MoHS). The APHL training ranged broadly and included basic bacteriology courses, influenza diagnostics, etc. Each of these trainings had its own challenges. Influenza diagnostics, for example, relied upon using ABI 7500 quantitative reverse-transcriptase polymerase chain reaction machines that were not well maintained, and it was extremely challenging to get reagents shipped in a timely fashion that maintained a cold chain. The APHL closed their offices in Sierra Leone on 26 February 2019.

The United States CDC began working in Sierra Leone during the 1970s, establishing a long-running research programme on Lassa fever.^3^ As part of the 2014–2016 EVD outbreak response, more than 700 CDC staff served on over 1000 deployments and, in 2015, a permanent CDC country office was established to focus on the Global Health Security Agenda.^4^ The CDC has established and supported training programmes ranging from field epidemiology training programme to an ecology and molecular diagnostics training programme with a university in Sierra Leone whose goal is to identify the animal reservoir of the Ebola virus.^5^ The CDC office in Sierra Leone has not published much information on their projects in Sierra Leone; however, their office remains open and runs surveillance, capacity building and epidemiology programmes. Programmes like the ‘*Creation of a national infection control programme in Sierra Leone*’ and the continuing field epidemiology training programme are indicative of the type of successful, ongoing engagements between CDC and Sierra Leone.^6^

China has a presence in Sierra Leone and the CCDC was a major international partner during the outbreak. The CCDC built a Biological Safety Level 3-capable laboratory space in combination with a hospital in Jui, a suburban neighbourhood to the east of Freetown and has been operating both since the early stages of the outbreak. Multiple teams of Chinese researchers and clinicians have rotated through the facilities and have maintained a consistent presence following the end of the EVD outbreak. In a recent press release, the director of the CCDC noted that more than 60 Chinese experts have been sent to Sierra Leone, and 30 Sierra Leoneans have studied and trained in China. CCDC supports ongoing national surveillance for Ebola, dengue fever, yellow fever, Zika and Lassa fever.^7^

Public Health England set out to renovate multiple Sierra Leone government laboratories in Sierra Leone, including the Connaught Hospital laboratory, the largest in Freetown. The PHE training programme focused on training national laboratory staff to international safety and quality standards, while teaching principles of molecular testing for Ebola virus and other high-consequence pathogens. The training was broken down into theory training and practical training. Theory training consisted of three sections: general information, a molecular theory course lasting two and half weeks and a molecular virology short course. Theory training occurred on multiple occasions, and the usual number of trainees at each session was approximately 15. Practical training lasted six weeks and was performed at three different government laboratories across Sierra Leone. At each site,^8,9^ trainees were trained and had supervised work experience and competence assessments performed by the PHE trainers. Additional support and training were given on alternative assays and platforms as well as maintenance support. Unfortunately, PHE has not published any reports on their training programmes, but it is the author’s opinion that the PHE trainers were of good quality and had developed a quality training programme. Public Health England is still supporting the renovated hospital laboratories. It is the author’s opinion that renovating hospital laboratories provides better return on investment than the construction or renovation of central or national public health laboratories in many circumstances, including in Sierra Leone.

## Challenges and future directions

Overall, there was little standardisation of programmes, materials or contact time with trainees between partner training programmes. Training materials and schedules occurred with very minimal input from the MoHS. Also, although two trainees may have similar certificates, the lack of standardisation of training programmes makes it difficult to compare skills between trainees. To this end, the author thinks it would be valuable for both the host country and partners to work together to standardise all training programmes and materials for training purposes as much as possible. The adage ‘practice makes perfect’ rings true in all molecular diagnostics training events and continued refreshers are extremely valuable if possible. When possible, the MoHS should require partners to use standardised procedures and assays. During the EVD outbreak, numerous organisations brought in their own proprietary assays, many of which were not commercially available. Trainees were trained on numerous assay platforms, and while this was necessary during the outbreak, it has been problematic during the post-outbreak capacity building phase. Staffing, purchasing, logistics and refresher training would all be easier to achieve with standard assays in place, used by all partners, as dictated by the MoHS.

Ideally, the MoHS should be in charge of: developing and providing training materials and standard operating procedures that are easily adaptable to all laboratories; providing individualised training assessments to guide personalised future training as well as laboratory operations refresher training on a regular basis. MoHS should also verify that implemented procedures are routinely performed. A comprehensive external quality assessment programme for all government laboratories would be an incredible accomplishment. This will more than likely happen very slowly, and there is always a risk that it may not happen at all. Therefore, it is recommended that partners organise with the MoHS to standardise and make the post-outbreak capacity building phase more efficient.

## MRIGlobal training history

As the EVD outbreak resolved and EVD cases decreased, the rapid diagnostic response also evolved. The MRIGlobal mobile diagnostic laboratory was one of the laboratories that was moved (from Moyamba to Lakka). Toward the end of the outbreak, there were far fewer blood samples being tested, and as the need for urgent response diminished, the focus turned to permanent transitioning of laboratories to the MoHS and training of the MoHS National Rapid Response Team.

MRIGlobal is an independent, not-for-profit organisation that performs aspects of laboratory design, operations, biosafety and security, research and diagnostics for government, academia and industry in the United States and internationally. MRIGlobal conducted training at the mobile diagnostic laboratory at CPHRL in Lakka during the outbreak. The duration of the training was six weeks and training components included didactic and kinesthetic training, laboratory simulations and continual refresher training based on molecular diagnostics testing for EVD. A total of eight graduates were trained using a wide variety of materials. Trainees also received quality assurance, quality control and biosafety training, which were rarely included in other partner training.

## MRIGlobal training programme

Disease surveillance systems in West Africa grapple with the problem of how to function, train and persist in resource-poor settings. It is vital for surveillance systems, especially surveillance systems in resource-poor settings, to increase capacity efficiently by building or repurposing infrastructure. However, often funding for infrastructure is limited and can be difficult to sustain; therefore, comprehensive training of professional staff is more likely to give a better return on investment.

With support from the United States Defense Threat Reduction Agency, and MRIGlobal, the Sierra Leone MoHS has developed a training programme to assist in disease surveillance in West Africa. The MRIGlobal molecular diagnostics training curriculum includes: PowerPoint lectures, hardcopy handouts and notes, textbooks, quizzes and exams, as well as all the physical training materials (pipettes, appropriate personal protective equipment, molecular laboratory equipment, biosafety cabinets, etc.) to fulfil an immersive molecular diagnostics (specifically EVD) training experience. The training programme utilises team mentoring (usually a team of two or three trainers) and supervision of trainees by subject matter experts, in which Sierra Leone MoHS staff are trained by MRIGlobal staff. Participants were given exit surveys throughout the training in 2015 and 2016 which showed a high degree of satisfaction with most aspects of the programme, including the length of the programme and the content (unpublished results). A key strength of the training programme is a true partnership approach, which utilises the use of onsite laboratory equipment to offer assorted training to Sierra Leone MoHS staff, and a team model for mentorship and supervision. The author believes the molecular diagnostics and disease surveillance training partnership established at the Sierra Leone CPHRL can be used as a model for sustainable capacity building and training in low-income and middle-income countries. Molecular diagnostics training included, but was not limited to, the following topics:

- Equipment overview, use, and maintenance
- Laboratory workflow process
- Pipetting
- Decontamination
- Personal protective equipment
- Biological waste disposal
- Introduction to RNA/DNA
- Introduction to virology
- Introduction to immunology
- Introduction to epidemiology
- Laboratory-acquired infections
- Quality management systems
- Specimen management

## Designing a locally sustainable programme

The MRIGlobal mobile diagnostic laboratory that served as an EVD diagnostic testing laboratory during the epidemic includes a sample extraction laboratory with multiple biosafety cabinets for sample inactivation and nucleic acid extraction, a reagent preparation space, and a quantitative real-time reverse-transcriptase polymerase chain reaction space. The purpose of the diagnostic training being held at the mobile diagnostic laboratory is to support the development of laboratory personnel and regional staff associated with the mobile diagnostic laboratory and to help integrate it into the existing CPHRL workflow.

Molecular diagnosis and surveillance require partnerships between laboratorians, public health experts and government officials. In order to adequately train personnel, numerous partnerships were established. Developing these partnerships served as the base for the programme at the Sierra Leone CPHRL.

MRIGlobal subject matter experts were very mindful to consider feasibility, sustainability and local relevance during the design of the training programme. This required aligning with national priorities and resources. The major topics of the diagnostics training programme developed are: safety protocols; laboratory orientation; reagent preparation; sample receipt and inactivation; nucleic acid extraction; quantitative real-time reverse-transcriptase polymerase chain reaction; data review, analysis and reporting; proficiency testing and targeting mentoring.

### Ethical considerations

This study followed all ethical guidelines for research involving no human participants.

## Discussion

Effective, operational laboratories are the pillar of effective clinical and public health systems, and are critical to the detection and diagnosis of infectious disease. In a recent publication, another international partner stated:

The absence of staff, stuff, space, and systems needed to detect outbreaks of infectious disease such as the recent Ebola epidemic in West Africa, and diagnose other medical conditions has underscored the need to not only set up diagnostic equipment in places where it is scarce, but also invest resources into training laboratory personnel. (p. 102)^10^

Laboratories worldwide suffer from scarcities of skilled or qualified staff. Payment for laboratory technicians and other categories of laboratory workers is lower than other specialties, and periodically delayed. Numerous times from 2014 to 2017 in Sierra Leone, government laboratory staff went unpaid for months due to the inability of the government to pay its workers. College-level and formal training opportunities are very limited or non-existent. A large proportion of laboratory staff are chosen without having the proper certificates, degrees or technical expertise necessary to carry out their responsibilities, resulting in systemic failures. Training students in diagnostic techniques is not an easy task. Expecting trainees to learn molecular diagnostic skills in short courses of two to six weeks is unreasonable and not sustainable. Even the best trainees require more than six weeks of training to become truly proficient, which is why refresher training or continued oversight is necessary for success. In order to truly make a sustainable difference regarding staff training and performance, organisations interested in training should be very conscious of whom they select for training, be prepared to provide as much refresher training as necessary and be able to provide some financial incentive or balance the training with the daily work tasks of laboratory staff.

Additionally, laboratory staff often lack access to adequate tools and supplies. Resource-poor laboratories often use obsolete technologies, expired reagents and improperly or uncalibrated equipment. The lack of equipment maintenance further erodes laboratory capabilities. Electricity instability in many low-income countries results in power surges or outages that damage equipment. Proper personal protective equipment is often lacking or compromised, resulting in hazardous work conditions for the staff. Funding organisations need to have equipment maintenance and replacement plans, as well as personal protective gear and consumable requirements, in place before a training programme begins.

In many low-income countries, adequate space is difficult to find. Many laboratories are located in small, cluttered spaces in hospitals. Often, laboratories consist of a single room, and operations meant to be done in separate spaces are done near one another. Many laboratories do not have a good water or electrical supply. Fuel for generators is expensive and, while useful to keep vital equipment running, is not sustainable.

Laboratories lacking trained staff, stuff and appropriate space often find it very difficult to develop robust systems. Quality, biosafety, accurate recording and reporting and a culture of maintenance are all critical laboratory functions; however, they are often not clearly understood or are under-prioritised. National guidelines and policies are often inadequate by international standards. Communication between Ministries of Health and international partners is often lacking. With Sierra Leone, as discussed above, numerous international organisations were training laboratory staff using a variety of different techniques and materials. Communication was very important to limit training overlap, trainee poaching and a variety of other potential misunderstandings.

Maintenance is difficult to instil, and without service technicians, eventually equipment reaches obsolescence. Rust and dirt are constant enemies of laboratory equipment, especially in non-climate-controlled environments. Performance skills of laboratory staff can also decline without consistent use or refresher training. Without active training, mentorship and quality management systems in the laboratory, performance can diminish. Both equipment and staff performance decline, due to lack of maintenance or skills usage, and are important considerations when establishing a training programme.

## Summary

Following the West Africa EVD outbreak, a high priority was placed on the training of staff and building or repurposing of infrastructure. MRIGlobal worked closely with the Sierra Leone MoHS to develop a sustainable, replicable training programme for diagnostics. With the proper prioritisation by the Sierra Leone MoHS and international partners, sustainable gains can be made in the area of clinical diagnostics, which will help mitigate future outbreaks. As stated previously, the author believes the molecular diagnostics and disease surveillance training partnership established at the Sierra Leone CPHRL can be used as a model for sustainable capacity building and training in low-income and middle-income countries. It is also the author’s opinion that long-term (10–20 year) sustainable engagement plans will be ultimately the most successful in Sierra Leone.
